# Could PTH/Ca Ratio Serve as a New Marker for Evaluating Bone Metabolism in Hemophilia Patients?

**DOI:** 10.3390/diagnostics15050638

**Published:** 2025-03-06

**Authors:** Tuba Ersal, Fazıl Çağrı Hunutlu, Vildan Gürsoy, Ezel Elgün, Şeyma Yavuz, İpek Dal Akkuş, İlayda Baş, Vildan Özkocaman, Fahir Özkalemkaş

**Affiliations:** 1 Division of Hematology, Department of Internal Medicine, Faculty of Medicine, Bursa Uludag University, Bursa 16059, Turkey; fazilhunutlu@gmail.com (F.Ç.H.); vildanterzioglu@hotmail.com (V.G.); elgunezel@hotmail.com (E.E.); seymayavuz2011@hotmail.com (Ş.Y.); vildanoz@uludag.edu.tr (V.Ö.); fahir@uludag.edu.tr (F.Ö.); 2 Department of Internal Medicine, Faculty of Medicine, Bursa Uludag University, Bursa 16059, Turkey; ipekdal@uludag.edu.tr (İ.D.A.); ilaydabas@uludag.edu.tr (İ.B.)

**Keywords:** bone mineral density, calcium, hemophilia, parathyroid hormone

## Abstract

**Background/Objectives:** Low bone mineral density (BMD) is common in hemophilia patients. Identifying high-risk patients for low BMD early is essential to prevent complications and reduce morbidity. The parathyroid hormone (PTH)/calcium (Ca) ratio is a cost-effective marker for predicting BMD, highlighting the need for routine screening and early intervention in this population. Hemophilia is a hereditary bleeding disorder caused by deficiencies in clotting factors VIII (hemophilia A) and IX (hemophilia B). Patients with hemophilia are at risk of low bone mineral density (BMD). This study aimed to evaluate the prevalence of low BMD, associated risk factors, and raise awareness regarding its significance in hemophilia patients. **Methods:** We retrospectively assessed bone metabolism in 62 hemophilia patients followed at our center. BMD was evaluated using dual-energy X-ray absorptiometry (DEXA). Additionally, serum levels of 25-OH-D3, alkaline phosphatase, PTH, Ca, phosphor, and creatinine were measured. The PTH/Ca, PTH/25-OH-D3, and Ca×25-OH-D3/PTH ratios were calculated. **Results:** The median age of the 62 patients with hemophilia included in the study (hemophilia A: 87.1%, hemophilia B: 12.9%) was 37 years (range: 21–66), and all were male. Of these patients, 67.7% (*n* = 42) had severe, 21% (*n* = 13) had moderate, and 11.3% (*n* = 7) had mild hemophilia. A total of 85.5% of patients were on factor prophylaxis, and 75.4% had a target joint. In laboratory analysis, the median 25-OH-D3 level was 13.4 µg/L and 75% patients had 25-OH-D3 deficiency. According to DEXA results, 62.9% had lower than normal BMD. When we divided the patients into normal and low BMD groups according to DEXA results, weight (*p* = 0.006), height (*p* = 0.024), factor levels (*p* = 0.004), PTH (*p* = 0.010), AST (*p* = 0.029), and PTH/Ca (*p* = 0.011) levels were statistically significantly different between the groups. The severity of the disease and the rate of receiving prophylaxis were higher in the group with low BMD (*p* = 0.015, *p* = 0.006, respectively). In multivariate analysis, PTH/Ca ratio and weight were found to be independent risk factors for BMD. A linear relationship was found between PTH/Ca ratio and BMD. The optimal cut-off value for PTH/Ca was 6.57, with a selectivity of 65% and specificity of 82%. When we divided the patients into groups according to the cut-off value of 6.57, we found that the probability of low BMD increased approximately 7-fold in the group with PTH/Ca > 6.57 (OR 7.045, 95% CI 1.485–33.42, *p* = 0.014). There was an inverse association between patient weight and low BMD (*p* = 0.043). **Conclusions:** Low BMD is a critical public health concern frequently observed in patients with hemophilia. The study highlights a high rate of low BMD and 25-OH-D3 deficiency in hemophilia patients, with the PTH/Ca ratio shown to be useful in predicting BMD. The PTH/Ca ratio is suggested as an accessible, cost-effective, and practical test for evaluating BMD in hemophilia patients.

## 1. Introduction

Hemophilia A and hemophilia B are rare, X-linked recessive congenital bleeding disorders caused by the absence or deficiency of coagulation factors VIII (FVIII) and IX (FIX), respectively. The disease is mostly transmitted from carrier women to their male children. Although the disease is very rare, it can also be seen in girls. In approximately 1/3 of the cases, the disease may occur with spontaneous de novo mutations without a family history [1]. Hemophilia A and hemophilia B constitute 85% and 15% of all patients with hemophilia, respectively. The clinical features of both types of hemophilia are similar. Hemophilias are a group of chronic diseases that mainly manifest with intra-articular (hemarthrosis) and intramuscular (hematoma) bleeding, and affect quality of life [2]. The severity of the disease is generally directly related to the degree of factor deficiency. Those with a factor level between 5 and 30% are called mild hemophilia, those with a factor level between 5% and ≥1% are called moderate hemophilia, and those with a factor level less than 1% are called severe hemophilia [3]. Factor replacement therapy involves the administration of FVIII and FIX concentrates to treat acute bleeding (on demand) or prophylactically to prevent bleeding. Regular preventive infusion of clotting factors to maintain factor levels above 1% in severe hemophilia reduces bleeding frequency, prevents related complications, and improves survival [4].

Advances in the monitoring and treatment of hemophilia have increased the life expectancy of patients with hemophilia. However, various comorbidities, particularly cardiovascular, metabolic, renal, and malignant diseases, are being encountered with increasing frequency. One of the chronic problems in hemophilia patients is that they are at risk for low bone mineral density (BMD). Osteoporosis in hemophilia is characterized by impaired bone microarchitecture along with reduced BMD. Many data have shown that low BMD starts at an early age and the risk of fractures due to minor traumas increases [5]. However, the pathogenesis of osteoporosis has not been fully elucidated, and is multifactorial [recurrent joint bleeding, decreased mobility or prolonged immobilization [6], nutritional deficiencies (especially vitamin D), FVIII and FIX deficiency, thrombin deficiency [7], changes in osteoblast and osteoclast activity [8].

The literature data on the link between hemophilia severity and decreased BMD are limited. Furthermore, it is not clear whether patients with hemophilia need routine bone mass monitoring; it may be recommended in patients with high risk or multiple risk factors [4].

The aim of this study was to investigate the frequency of osteopenia/osteoporosis in patients with hemophilia, to examine the risk factors and to draw attention to the relationship between hemophilia severity and low bone density.

## 2. Materials and Methods

Sixty-two hemophilia patients were followed up at the Department of Adult Hematology at Uludağ University, Bursa, between January 2020 and August 2024. BMD was assessed using dual-energy X-ray absorptiometry (DEXA), with additional height, weight, and BMI measurements. Laboratory tests included serum 25-hydroxy vitamin D (25-OH-D3), calcium, phosphor, ALP, PTH, AST, ALT, creatinine, and factor levels (FVIII for hemophilia A, and FIX for hemophilia B), along with hepatitis serologies. The PTH/Ca, PTH/25-OH-D3, and Ca×25-OH-D3/PTH ratios were calculated.

Serum calcium, phosphor, creatinine, ALP, AST, and ALT levels were measured on an ARCHITECT C-16000 analyzer (Abbott Diagnostics, Abbott Park, IL, USA), and 25-hydroxy vitamin D and PTH levels were measured on an ARCHITECT İ-2000 SR Immunoassay analyzer (Abbott Diagnostics, Abbott Park, IL, USA).

FVIII and FIX were studied with one stage clotting assays using Siemens Coagulation Factor Deficient Plasma commercial kits (Siemens Healthcare Diagnostics Products, Germany) on a Sysmex CN-6000 analyzer (Sysmex, Kobe, Japan).

For patients under 50 yr, DEXA results were classified by World Health Organization criteria: normal if the *T* score was greater than −1, osteopenia if between −1 and −2.5, and osteoporosis −2.5 or lower [9]. The International Society for Clinical Densitometry guidelines suggest using the *Z* score for men under 50 yr; a *Z* score of −2.0 or lower is “lower than expected for age,” and greater than −2.0 is “within the expected range for age” [10]. The term “low BMD” refers to osteopenia/osteoporosis or values lower than expected for age.

The 2018 Guidelines of the Turkish Society of Endocrinology and Metabolism define serum 25-OH-D3 levels > 30 ng/mL as sufficient, 20–30 ng/mL as insufficient, <20 ng/mL as deficient, and <10 ng/mL as severely deficient.

### Statistical Analyses

Statistical analyses were conducted using the IBM Statistical Package for the Social Sciences Statistics for Windows v29.0 (IBM Corp., Armonk, NY, USA). Continuous variables are presented as means and standard deviations for normally distributed data, and as medians with minimum–maximum values for nonnormally distributed data. Student’s *t* test or Mann–Whitney *U* test was used to compare continuous variables between groups. Categorical variables were compared using the chi-square test. Receiver operating characteristic (ROC) curve analysis was used to determine the optimal cut-off values for the PTH/Ca ratio in relation to low BMD. Multivariate binary logistic regression analysis with the backward LR method was used for factors with a univariate *p* value < 0.2, with statistical significance set at *p* < 0.05.

## 3. Results

Among the 62 patients (54 [87.1%] with hemophilia A and 8 [12.9%] with hemophilia B), 67.7% (*n* = 42) had severe, 21% (*n* = 13) had moderate, and 11.3% (*n* = 7) had mild hemophilia. Median age was 37 (range: 21–66), and 11 patients were >50 yr. All patients were male, with 85.5% receiving factor prophylaxis. Only one patient had an inhibitor, and was therefore using a bypassing agent. A target joint was identified in 75.4% of the patients. In the overall cohort, the median vitamin D level was 13.4 µg/L (4.4–51.6), with approximately 75% of patients with vitamin D deficiency (<20 µg/L), and 38% with severe vitamin D deficiency (<10 µg/L). Anti-HCV positivity was detected in approximately 14% (*n* = 8) of the patients. HCV-RNA was negative in four of these patients who had never received antiviral treatment. Three patients were HCV-RNA negative with antiviral treatment received in the past, and were in drug-free follow-up. Only one patient had HCV-RNA copy number 5,369,855 and received active HCV antiviral treatment (Glecaprevir combined with Pibrentasvir) for about 6 weeks. The clinical and laboratory characteristics of the study participants are summarized in Table 1.

DEXA evaluations revealed that 37.1% of patients had normal BMD, while 62.9% had low BMD. Median DEXA *T* scores for the entire cohort were as follows: femoral neck, −1.1 (range: −4.4 to 2.6); total hip, −1.0 (range: −4.5 to 2.1); and total vertebra, −0.70 (range: −3 to 2.6). Corresponding *Z* scores were −0.80 (range: −3.9 to 3.1) for the femoral neck, −0.8 (range: −4.3 to 2.1) for the total hip, and −0.70 (range: −3 to 2.6) for the total vertebra. A summary of the DEXA scores is provided in Table 2.

In patients under 50 yr of age, 17 (33.3%) had normal BMD, while 34 (66.7%) presented with low age-adjusted BMD. Among the eleven patients aged over 50 yr, six (54.5%) had normal BMD, three (27.2%) were classified as osteopenic, and two (18.1%) were osteoporotic.

When patients were categorized based on DEXA results into groups with normal and low BMD, statistically significant differences were observed between the groups regarding weight (*p* = 0.006), height (*p* = 0.024), factor levels (*p* = 0.004), PTH levels (*p* = 0.010), AST levels (*p* = 0.029), and the PTH/Ca ratio (*p* = 0.011). No significant differences were found for other variables. Patients with low BMD had higher disease severity and a higher rate of receiving prophylactic treatment compared with those with normal BMD (*p* = 0.015 and *p* = 0.006, respectively; Table 3).

**Figure 1:**
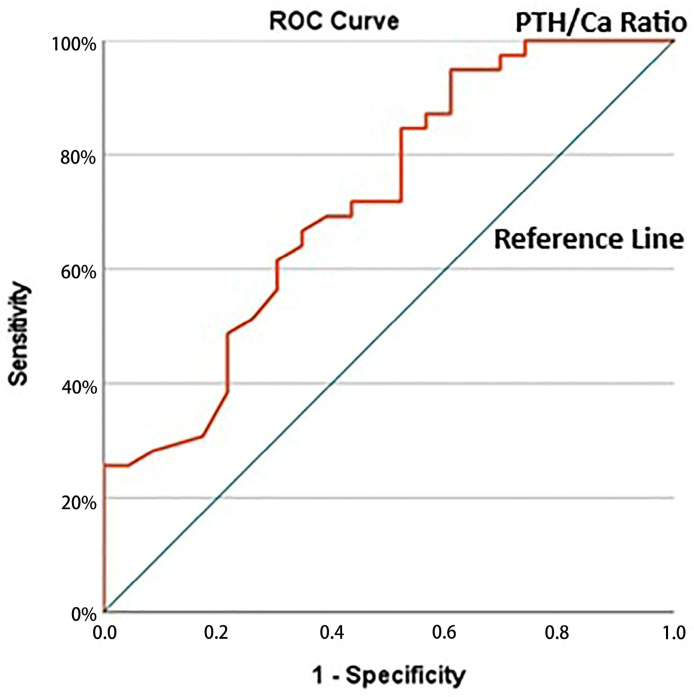
ROC curve analysis of PTH/Ca ratio.

In the ROC analysis, the optimal cut-off value for the PTH/Ca ratio, determined using the Youden index, was 6.27 (Figure 1). This cut-off yielded an AUC of 0.731 (95% CI, 0.583–0.880; *p* = 0.009) with a sensitivity of 65% and a specificity of 82% (Table 4). In the ROC analysis, the cut-off factor level determined by the Youden index was 1.27%, with an area under the curve (AUC) of 0.719 (95% CI, 0.585–0.852; *p* = 0.004). At this cut-off, sensitivity was 85%, and specificity was 48% (Table 4).

Table 5 summarizes the univariate and multivariate analyses for BMD. In the multivariate analysis, weight and the PTH/Ca ratio emerged as independent risk factors for low BMD (*p* = 0.043 and *p* = 0.014, respectively). The PTH/Ca ratio was directly proportional to low BMD. A higher PTH/Ca ratio was associated with an approximately sevenfold increase in the likelihood of low BMD (OR, 7.045; 95% CI, 1.485–33.42; *p* = 0.014). Additionally, an inverse relationship was observed between weight and low BMD (*p* = 0.043).

## 4. Discussion

The results of this study, in which we evaluated BMD in patients with hemophilia, show that 62.9% of our patients with hemophilia had low BMD. The serum PTH/Ca ratio, which was measured simultaneously with DEXA, and patient weight were found to be independent risk factors for low BMD. To our knowledge, our study is the first study in which PTH/Ca ratio was evaluated for BMD in hemophilia patients.

Studies from various countries have reported different prevalent rates for low BMD in hemophilia patients. In a very recent study, Ransmann et al. reported a rate of 63.1%, which is very close to the rate in our study [11]. A study in Turkey, for example, reported a low BMD rate of 26.8% [12], and a study from China reported 41.5% [13]. In contrast, Gerstner et al. [14] described higher rates in a cohort of 30 patients, with 43% exhibiting osteopenia and 27% osteoporosis. These differences may be attributable to age, disease severity, treatment strategies, or varying definitions of low BMD in patients with hemophilia.

FVIII and FIX have been shown to play important roles in bone hemostasis [15,16], with a clear correlation observed between the severity of hemophilia and changes in bone [17]. Patients with severe hemophilia are at an increased risk for fractures [18]. Additionally, studies have demonstrated that hemophiliac mice exhibit lower BMD and abnormal bone structure compared with normal mice [15,19]. Studies investigating the molecular mechanisms of FVIII or FIX deficiency leading to osteoporosis have shown that the RANK/RANKL/OPG signaling pathway plays a role in the development of osteoporosis in patients with hemophilia [18,20,21]. Receptor activator of nuclear factor kappa-B ligand (RANKL) binds to its receptor RANK and promotes osteoclastogenesis, leading to increased bone resorption [22]. Osteoprotegerin (OPG) attenuates osteoclastogenesis through competitive binding with RANKL [23]. This pathway is essential for maintaining bone turnover homeostasis. The FVIII–VWF complex has been found to play a direct role in bone remodeling by binding to RANKL and OPG, inhibiting RANKL-induced osteoclastogenesis, and enhancing the inhibitory effects of OPG on osteoclasts, thereby promoting osteogenesis [24]. This hypothesis is further supported by studies showing thrombin receptors on osteoblasts [16] and the inhibitory effect of factor VIII on osteoclastogenesis [24]. Consistent with the literature, our study found a linear relationship between factor levels and BMD [25]; however, this association lost significance in the multivariate analysis.

Deficiency in 25-OH-D3 increases the risk of osteoporosis, osteopenia, and bone fragility in normal individuals [26]. Although few studies have examined the relationship between 25-OH-D3 and BMD in hemophilia patients, the results have been inconsistent. One study suggested that low 25-OH-D3 levels in hemophilia patients may be associated with osteoporosis [14]. In a series of 61 patients with hemophilia, no significant correlation was observed between BMD and 25-OH-D3 [27]. Another recent study indicated that 25-OH-D3 deficiency was significantly associated with reduced total hip and femoral neck BMD and increased fracture risk, also suggesting that 25-OH-D3 plays a role in the causal relationship between bone remodeling and BMD in patients with osteoporosis or fractures. Evaluating 25-OH-D3 status was deemed essential for individuals at risk of osteoporosis or fractures [28]. In our study, 75% of patients had deficient serum 25-OH-D3 levels, a comparatively high rate, with a similar result observed in a study conducted in Turkey (79.3%) [27]. However, in the study by Wu et al. [13], no 25-OH-D3 deficiency (defined as a level <30 µg/L) was detected in any patient. In another study, 25-OH-D3 deficiency and insufficiency were found in 27% and 67% of patients, respectively [14]. Nonetheless, our study found no statistically significant relationship between serum 25-OH-D3 and BMD.

The 25-OH-D3 and PTH operate in a feedback cycle where they regulate each other. In cases of 25-OH-D3 deficiency, serum PTH levels increase, leading to secondary hyperparathyroidism. PTH subsequently increases calcium metabolism from bones and stimulates bone turnover. However, for unknown reasons, this secondary hyperparathyroidism does not occur uniformly in all patients with mild vitamin D deficiency [26]. As anticipated, a negative correlation was found between PTH and 25-OH-D3 in our study (Pearson correlation, *p* = 0.002, coefficient = −0.408). PTH levels were higher in patients with low BMD than in those with normal BMD. Calcium levels were slightly higher in patients with low BMD than those with normal BMD, but this difference was not statistically significant. This finding led us to believe that a sufficient serum calcium level could not be achieved, despite elevated PTH levels. For the first time in evaluating bone metabolism in patients with hemophilia, we applied the PTH/Ca ratio, which was significantly higher in patients with low BMD. When the optimal cut-off value of this ratio to predict low BMD was set at 6.57, the likelihood of low BMD increased sevenfold in patients with a PTH/Ca ratio above this value. This finding suggests that the PTH/Ca ratio may serve as an inexpensive and practical test for routinely predicting low BMD.

Low BMI is a known risk factor for fractures in postmenopausal women [29]. Previous studies on the relationship between BMI and BMD in hemophilia patients have produced mixed results, with some reporting a positive correlation and others reporting no association [12,25]. In our study, weight emerged as an independent risk factor for low BMD and was inversely associated with BMD, although no significant relationship was found between BMI and BMD.

Hepatitis C virus-associated hepatitis and liver disease may contribute to low bone mass [19]. Conflicting perspectives exist in the literature on the relationship between HCV infection and low BMD. While Masaki and Katsarou suggested that chronic inflammation due to hepatitis C may predispose patients to low BMD, Linari et al. [30,31,32] found no association between the two. In our study, no relationship was found between HCV and BMD.

Hemophilia patients develop hemarthrosis, cartilage damage, and hemophilic arthropathy as a result of recurrent joint bleeding [33]. Several studies have documented an inverse relationship between the severity of arthropathy and BMD [14,31]. When we analyzed the relationship between BMD and the presence and number of target joints, as well as the presence of target joints in the lower extremities and BMD, we found no significant relationship between the presence and the number of target joints and BMD. Although target joints in the lower extremities were more common in patients with low BMD, this was not statistically significant.

This study has some limitations, including its retrospective, single-center design and the inability to measure urine calcium levels, which are related to PTH and calcium, due to its retrospective nature.

## 5. Conclusions

Low BMD is frequently observed, particularly in individuals with severe hemophilia. Early detection is crucial in hemophilia patients because of the high risks of falling, fractures, and bleeding due to low BMD, the challenges of potential surgical interventions, high morbidity and mortality rates, and prolonged hospitalization. Therefore, early screening is recommended. However, radiation exposure, albeit minimal, may limit the feasibility of frequent DEXA scans in children, and some patients may not be physically able to undergo DEXA. In such cases, the PTH/Ca ratio could serve as an early warning test for low BMD.

## Figures and Tables

**Table 1:** Demographic, clinical, and laboratory characteristics of patients.

Variable (Median, Min–Max)	Hemophilia A *n* = 54 (87.1%)	Hemophilia B *n* = 8 (12.9%)	All Patients *n* = 62 (100%)
Age, years	37 (21–66)	36 (23–62)	37 (21–66)
Weight, kg	80 (53–135)	75 (54–120)	79 (53–135)
Height, cm	170 (150–190)	170 (167–187)	170 (150–190)
Factor levels, %	0.25 (0.02–12.3)	2.31 (0.9–19.7)	0.3 (0.02–19.7)
Disease severity			
Severe (<1%)	41 (75.9%)	1 (12.5%)	42 (67.7%)
Moderate (1–5%)	8 (14.8%)	5 (62.5%)	13 (21%)
Mild (>6–40%)	5 (9.3%)	2 (25%)	7 (11.3%)
Inhibitor			
Yes	1 (1.9%)	0 (0%)	1 (1.6%)
No	53 (98.1%)	8 (100%)	61 (98.4%)
Current treatment			
Prophylaxis	48 (88.9%)	5 (62.5%)	53 (85.5%)
When bleeding occurs	6 (11.1%)	3 (37.5%)	9 (14.5%)
Target joint			
Yes	41 (75.9%)	5 (62.5%)	46 (75.4%)
No	13 (24.1%)	3 (37.5%)	15 (24.6%)
25-OH-D3, µg/L	14.3 (4.6–51.6)	9.4 (4.4–19.9)	13.4 (4.4–51.6)
Total protein, g/L	74 (66–80)	76 (71–79)	74 (66–80)
Ca, mg/dL (mean, *SD*)	9.0 (8.3–10.2)	9.1 (8.1–9.5)	9.1 (8.1–10.2)
P, mg/dL (mean, *SD*)	3.25 (1.8–4.8)	2.65 (2.1–4.1)	3.2 (1.8–4.8)
ALP, U/L	96 (38–167)	85 (56–139)	90 (38–167)
PTH, ng/L	59.7 (10.1–140)	47.7 (22.2–117.4)	59.1 (10.1–140)
AST, U/L	20 (11–57)	18.5 (14–53)	20 (11–57)
ALT, U/L	19 (6–108)	15.5 (12–89)	19 (6–108)
Creatinine, mg/dL	0.81 (0.57–1.54)	0.77 (0.62–1.06)	0.8 (0.57–1.54)
Anti-HCV positivity	7 (14.3%)	1 (12.5%)	8 (14%)

Abbreviations: 25-OH-D3, 25 hydroxy vitamin D; ALP, alkaline phosphatase; ALT, alanine aminotransferase; AST, aspartate aminotransferase; PTH, parathyroid hormone.

**Table 2:** DEXA scores according to *T* and *Z* scores.

Variable (Median, Min–Max)	Hemophilia A *n* = 54	Hemophilia B *n* = 8	All Patients *n* = 62
Femoral neck *T* score ^a^	−1.25 (−4.4 to 1.6)	0.65 (−2.4 to 2.6)	−1.1 (−4.4 to 2.6)
Femoral neck *Z* score ^b^	−0.95 (−3.9 to 2)	0.65 (−2.3 to 3.1)	−0.80 (−3.9 to 3.1)
Femur total *T* score ^a^	−1.0 (−4.5 to 1.2)	1.05 (−2.5 to 2.1)	−1.0 (−4.5 to 2.1)
Femur total *Z* score ^b^	−0.85 (−4.3 to 1.6)	1.15 (−2.4 to 2.2)	−0.8 (−4.3 to 2.1)
Total vertebral *T* score ^a^	−0.75 (−2.8 to 2.6)	−0.15 (−3 to 1.6)	−0.70 (−3 to 2.6)
Total vertebral *Z* score ^b^	−0.70 (−2.8 to 2.6)	−0.15 (−3 to 2.1)	−0.70 (−3 to 2.6)
DEXA			
Normal	18 (33.3%)	5 (62.5%)	23 (37.1%)
Low	36 (66.7%)	3 (37.5%)	39 (62.9%)

Abbreviation: DEXA, dual-energy X-ray absorptiometry. ^a^ Patients over 50 yr of age. ^b^ Patients under 50 yr of age.

**Table 3:** Patient characteristics according to BMD.

Variable (Median, Min–Max)	Normal BMD *n* = 23, %	Low BMD *n* = 39, %	*p*
Age, years	28 (21–62)	38 (22–66)	0.089 ^c^
Weight, kg	88 ± 17.6	77 ± 12.1	**0.006** ^b^
Height, cm	175 (160–190)	170 (150–189)	**0.024** ^c^
BMI, g/cm^2^	27.5 (22–47)	26.3 (17–37)	0.265 ^c^
Factor level, %	0.80 (0.18–19.7)	0.24 (0.02–6.15)	**0.004** ^c^
Disease severity			
Severe	12 (52.2%)	30 (76.9%)	**0.015** ^a^
Moderate	5 (21.7%)	8 (20.5%)	
Mild	6 (26.1)	1 (2.6%)	
Current treatment			
Prophylaxis	16 (69.6%)	37 (94.9%)	**0.006** ^a^
When bleeding occurs	7 (30.4%)	2 (5.1%)	
Anti-HCV positivity			
Yes	2 (9.1%)	6 (15.4%)	0.439 ^a^
No	20 (90.9%)	31 (79.5%)	
Presence of the target joint			
Yes	15 (65.2%)	31 (79.5%)	0.215 ^a^
No	8 (34.8%)	8 (20.5%)	
Number of target joints			
One	6 (26.1%)	5 (12.8%)	0.104 ^a^
Multiple	9 (39.1%)	26 (66.7%)	
Presence of the target joint in the lower extremity			
Yes	12 (52.2%)	29 (76.3%)	0.052 ^a^
No	11 (47.8%)	9 (23.7%)	
25-OH-D3, µg/L	10.8 (4.4–51.6)	14.8 (4.6–41.5)	0.785 ^c^
Ca, mg/dL (mean, *SD*)	9.05 ± 0.44	9.1 ± 0.27	0.300 ^b^
P, mg/dL (mean, *SD*)	3.3 ± 0.63	3.2 ± 0.62	0.167 ^b^
ALP, U/L	88.5 (53–126)	95 (38–167)	0.706 ^c^
PTH, ng/L (mean, *SD*)	47.5 ± 23.2	64.1 ± 25.3	**0.010** ^b^
AST, U/L	21 (13–53)	19 (11–57)	**0.029** ^c^
ALT, U/L	22 (10–89)	17 (6–108)	0.072 ^c^
Creatinine, mg/dL	0.82 (0.57–1.54)	0.80 (0.62–1.10)	0.184 ^c^
GFR	118 (48–132)	115 (86–135)	0.646 ^c^
Ca×25-OH-D3/PTH	2.33 (0.70–21.4)	2.01 (0.38–10.2)	0.357 ^c^
PTH/Ca (mean, *SD*)	5.25 ± 2.60	7.09 ± 2.83	**0.011** ^b^
PTH/25-OH-D3	4.04 (0.48–12.9)	4.67 (0.87–22.7)	0.348 ^c^

Abbreviations: 25-OH-D3, 25-hydroxy vitamin D; ALP, alkaline phosphatase; ALT, alanine aminotransferase; AST, aspartate aminotransferase; BMI, body mass index; Ca, calcium; GFR, glomerular filtration rate; HCV, hepatitis C virus; P, phosphorus; PTH, parathyroid hormone. ^a^ Chi-square test (*χ*^2^). ^b^ Independent sample *t* test. ^c^ Mann–Whitney *U* test. Bold value: Statistically significant.

**Table 4:** Predictive value analysis of variables for distinguishing low BMD.

Variable	AUC	95% CI	Cut-Off	Sensitivity	Specificity	*p* Value
Factor level	0.719	0.585–0.852	1.27	85	48	**0.004**
PTH/Ca	0.731	0.583–0.880	6.57	65	82	**0.009**

Abbreviations: 95% CI, 95% confidence interval; AUC, area under the curve. Bold value: Statistically significant.

**Table 5:** Univariate and multivariate analysis for BMD.

	Univariate Logistic Regression			Multivariate Logistic Regression		
Risk Factors	OR	95% CI	*p*	OR	95% CI	*p*
Age, yr	1.036	0.988–1.087	0.146			
Height, cm	0.930	0.870–0.994	0.033			
Weight, kg	0.948	0.910–0.989	0.013	0.945	0.895–0.998	**0.043**
BMI, g/cm^2^	0.931	0.832–1.041	0.210			
Ca, mg/dL	1.518	0.328–7.031	0.594			
P, mg/dL	0.641	0.262–1.567	0.330			
Creatinine, mg/dL	0.082	0.003–2.536	0.153			
GFR	0.996	0.969–1.023	0.746			
ALP, U/L	1.007	0.986–1.029	0.488			
PTH, ng/L	1.036	1.005–1.068	0.023			
25-OH-D3, µg/L	0.997	0.949–1.047	0.896			
Factor group (≤1.27, >1.27)	0.198	0.06–0.655	0.008	0.251	0.055–1.140	0.073
PTH/25-OH-D3	1.099	0.955–1.265	0.339			
Ca×25-OH-D3/PTH	0.869	0.736–1.027	0.128			
PTH/Ca group (≤6.57, >6.57)	7.467	1.84–30.27	0.005	7.045	1.485–33.42	**0.014**

Abbreviations: 95% CI, 95% confidence interval; ALP, alkaline phosphatase; BMI, body mass index; Ca, calcium; GFR, glomerular filtration rate; OR, odds ratio; P, phosphorus; PTH, parathyroid hormone. Bold value: Statistically significant.

## Data Availability

The datasets used and/or analyzed during the current study are available from the corresponding author upon reasonable request.
